# Perceptions of Social Mobility: Development of a New Psychosocial Indicator Associated with Adolescent Risk Behaviors

**DOI:** 10.3389/fpubh.2015.00062

**Published:** 2015-04-16

**Authors:** Miranda Lucia Ritterman Weintraub, Lia C. H. Fernald, Nancy Adler, Stefano Bertozzi, S. Leonard Syme

**Affiliations:** ^1^Public Health Program, Touro University California, Vallejo, CA, USA; ^2^School of Public Health, University of California Berkeley, Berkeley, CA, USA; ^3^Department of Psychiatry, University of San Francisco, San Francisco, CA, USA

**Keywords:** adolescent health, social gradient, risk behaviors, international health, socioeconomic factors, social mobility

## Abstract

Social class gradients have been explored in adults and children, but not extensively during adolescence. The first objective of this study was to examine the association between adolescent risk behaviors and a new indicator of adolescent relative social position, adolescent “*perceived social mobility*.” Second, it investigated potential underlying demographic, socioeconomic, and psychosocial determinants of this indicator. Data were taken from the 2004 urban adolescent module of *Oportunidades*, a cross-sectional study of Mexican adolescents living in poverty. *Perceived social mobility* was calculated for each subject by taking the difference between their rankings on two 10-rung ladder scales that measured (1) projected future social status and (2) current subjective social status within Mexican society. Adolescents with higher *perceived social mobility* were significantly less likely to report alcohol consumption, drinking with repercussions, compensated sex, police detainment, physical fighting, consumption of junk food or soda, or watching ≥4 h of television during the last viewing. They were significantly more likely to report exercising during the past week and using a condom during last sexual intercourse. These associations remained significant with the inclusion of covariates, including parental education and household expenditures. Multiple logistic regression analyses show higher *perceived social mobility* to be associated with staying in school longer and having higher perceived control. The present study provides evidence for the usefulness of *perceived social mobility* as an indicator for understanding the social gradient in health during adolescence. This research suggests the possibility of implementing policies and interventions that provide adolescents with real reasons to be hopeful about their trajectories.

## Introduction

### Social gradient in health during adolescence

There is substantial evidence of a social gradient in many measures of physical and mental health among adults (1, 2) and young children (3, 4). Less is known about the associations between social position and health during adolescence, generally defined as individuals between the ages of 12 and 22, and existing findings are inconsistent (5–7). Assessment of subjective status has provided evidence for negative social gradients in health during adolescence for some physical, psychological, and behavioral health indicators, including overweight/obesity (8, 9), self-rated health (10), depressive symptoms (9, 11), and substance use (12, 13). Other studies have shown “equalization” occurring during adolescence, in which class-based differences that exist during childhood disappear during adolescence only to reemerge in adulthood (6).

Various explanations have been offered for the relative absence of an effect of social class on adolescent health: First, adolescent class identity may be less influenced by differential access to material resources (e.g., income, occupational grade and educational attainment level), and more influenced by social processes associated with social position such as national educational systems, meritocratic structure, redistributive policies, peer structures (14), cultural norms and values, and future expectations (15). Second, while household and parental indicators of SES may be useful proxies for the social status of infants and younger children, they may be less appropriate in assessing adolescent social position and social and economic resources; adolescents may already have attained a different social position than that of their parents, such as a different educational attainment level or occupational grade (13). Adolescents are in a transition between being defined by their parents’ social position and by their own. It is possible that adolescent social status may be influenced not only by the socioeconomic status of the family of origin and current status, but also by projections of a potential future socioeconomic trajectory (15), yet no studies to date have examined the associations between adolescent *perceived* class identify and health.

### Intergenerational social mobility and adolescent health

**Figure 1:**
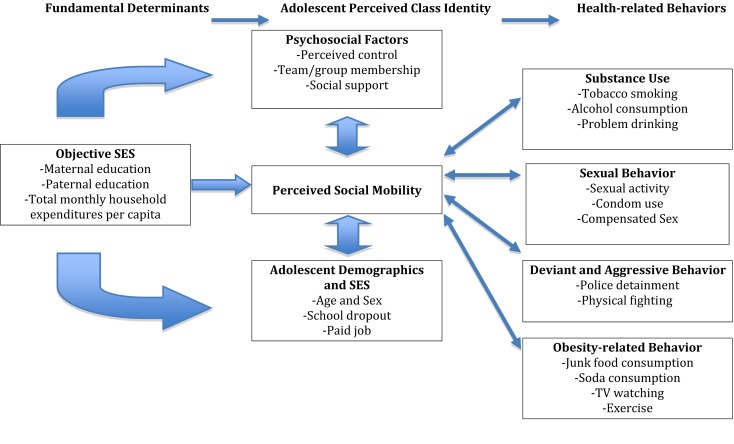
**Theoretical model of associations between perceived social mobility and socioeconomic status, demographic characteristics, psychosocial factors, and health-related behaviors**.

“Social mobility” has been defined as a shift made by individuals from one level of social status to another within a given social hierarchy. Adolescents’ evaluations of their socioeconomic position may not only consist of a cognitive averaging of external measures of their current socioeconomic status, but may also involve additional factors that could affect their perceptions of their future opportunities (Figure 1). These factors, which may be associated with social disadvantage, include demographic characteristics, such as dropping out of school, developmental shifts, psychosocial factors involving social relationships (e.g., network support), and psychological resources (e.g., mastery) (10).

Change in social standing can occur between and within generations (16). Intergenerational social mobility involves the social status of the target person and their parents and is measured by parent–child differences in income, educational attainment, and/or occupation. Several studies have found upward social mobility among adolescents and young adults in relation to their parents to be associated with better self-reported health (17), lower tobacco smoking (18–20), alcohol consumption (19, 20), consuming a high fat diet (19, 20), and eating sweets each day (19), and with a higher likelihood of being physically active (19). Some studies found no association between upward social mobility and smoking prevalence (20), alcohol consumption (20), and body mass index (21).

Attempts to measure the association between intergenerational mobility and health and risk behaviors among adolescents and young adults present numerous methodological complications, which may explain the pattern of inverse trends (17–19), or mixed results or no association (19–21) found in both cross-sectional and prospective cohort studies. First, measures of intergenerational mobility during adolescence and early adulthood should be regarded as provisional, since young participants, as young as 16–23 years (17–21) may still be attaining their education or establishing a career path; their socioeconomic trajectory from childhood to adulthood may not yet be determined. A second limitation is that indicators of parental and adolescent socioeconomic status can be incomparable. There is a range of ways to calculate intergenerational social mobility, involving both the SES of the parent/legal guardian and that of the adolescent or young adult. Some studies attempt to calculate intergenerational social mobility by classifying both the parent and the young participant into the same social status categories (e.g., non-university versus university or manager/professional versus non-manager/professional) (18, 21). This is a complicated issue. For example, if a participant is in school and does not yet have an occupation, they may be asked to state the occupation for which they are studying (21). This may not be relevant for many adolescents and young adults, as secondary education and some college degrees do not prepare individuals for a specific occupation. Other studies may use completely distinct social status categories or indicators. For example, one study calculated intergenerational occupational social mobility by comparing the occupation of the parent (upper white collar, lower white collar, blue collar or farmer) to the educational achievement and type of educational training of the young participant (e.g., vocational or not; school dropout or not) (19). When intergenerational social mobility is determined using different measures of social status, the estimated difference between the two social positions is not directly comparable (22).

### Present study

This paper presents data on a new adolescent psychosocial measure, developed to overcome the methodological limitations of standard measures of intergenerational social mobility and to examine the association between anticipated intergenerational social mobility and health-related behaviors during adolescence. We assess the association between adolescent *perceived social mobility* and twelve risk behaviors. We further explore the extent to which these associations can be explained by conventional measures of socioeconomic position, such as maternal and paternal education and total monthly household expenditures per person. We also investigate potential demographic, socioeconomic and psychosocial factors involved in the process of anticipating upward mobility versus stagnation or lower future status. The specific aims of the study are: (1) to establish whether *perceived social mobility* is associated with adolescent risk behaviors; and (2) to identify socioeconomic, demographic, and psychosocial factors that may determine adolescent *perceived social mobility*. Findings may inform the discussion of how to reduce health inequities in adolescence and early adulthood.

## Materials and Methods

### Procedure (study design and sampling)

The analyses use data gathered in 2004 for the evaluation of a poverty alleviation program in Mexico. All data were collected using an audio-computer assisted self-interview system, supplemented with a socioeconomic household questionnaire. The survey included 157 urban (defined as having 50,000–1 million inhabitants) towns in seven states in Mexico. Households were selected first, using census data for all census tracts. Areas with 500 or more eligible households were identified, and a sample of those with the highest density of eligible households was selected and then matched to comparison areas for evaluation purposes. Following this process, a random set of census tracts was identified within the areas with probability proportional to size. From this sample of 204 urban areas, a sub-set of 157 areas was selected for the adolescent risk behavior component. Up to three visits were made to each household in these areas to collect data on household SES as well as data on adolescent risk behavior. A total of 7900 adolescents, aged 12–22 years of age, were identified in this way. Of this group, 6929 (75%) had complete questionnaire, parental, household and neighborhood data.

We used data from the general household survey on household and parental SES as well as data from the adolescent survey. The survey included adolescent objective and subjective indicators of social position (current and future subjective social status, school dropout status, paid job), adolescent psychosocial characteristics (perceived control, team or group membership, social support), and adolescent demographics (age, sex). Adolescents who were married (*n* = 212), had children (*n* = 788), or were outside of the age range of 12–22 years (*n* = 2) did not meet our study criteria and were excluded from analysis. Further, adolescents missing data on perceived social mobility (*n* = 564), school dropout status (*n* = 100), paid work (*n* = 47), and group membership (*n* = 27) were excluded from the final sample. Our final sample for this analysis included 5189 adolescents (75%) of the original sample). We compared the adolescents with and without social mobility data. Those missing data were more likely to be older by 2.6 months on average, to have dropped out of school, to have parents with less than a primary education, to have lower perceived control and less social support, to be more likely to have used a condom during last sexual intercourse, to have watched over 4 h of television during their last viewing, and to be less likely to exercise. There were no significant differences by sex, paid job, household expenditures, team or group membership, substance-related behaviors, sexual activity, compensated sex, deviant behaviors, and diet.

The study was approved by the Research Committee at the National Institute of Public Health in Mexico, and by the Committee on the Protection of Human Subjects at the University of California at Berkeley. Participants were invited to take part in the 2004 study after receiving a detailed explanation of the survey procedures and signing an informed consent declaration. If the adolescent was under 16 years of age, parents were asked to provide consent and the adolescent was asked to provide assent.

### Measures

#### “Perceived social mobility” (adolescent self-report)

A modified version of the subjective social status (SSS) Scale-Youth Version was completed (9). Several 10-rung ladders were depicted, one of which was the standard SSS scale, while the others were new for this study. For all of the ladder scales, the top represents those with the highest ranking, the richest households, and the bottom represents those with the lowest ranking, the poorest households. The standard SSS question asked adolescents to make a relative comparison on the rich-to-poor ladder scale of their current household with “all households in Mexico.” A second question asked adolescents to think of the family they will have in the future (i.e., spouse and children), and to make a prediction of how their future nuclear household will compare with “all households in Mexico.” No time period in the future was specified.

The authors calculated *perceived social mobility* within the society as the difference in rank between anticipated future social position and perceived current social position, within the society. The scores could range from −9 to 9. Those who reported an equivalent rank or a rank of one or more steps lower in the future compared to the current ladder scale (0 to −9) were classified as “*stable or downwardly mobile*,” while those who reported a higher rank (1–9) were classified as “*upwardly mobile*.”

#### Sociodemographic covariates (adolescent self-report)

Adolescents provided data on age (continuous) and sex (male/female).

#### Objective adolescent socioeconomic position (adolescent self-report)

Adolescents were asked whether they had dropped out of school and whether they currently have a paid job.

#### Objective parental and household socioeconomic position (from parents’ survey)

##### Maternal and paternal education

Maternal and paternal education were represented by dichotomous variables: “primary education or less,” “secondary and above.”

##### Total monthly household expenditure

Total monthly household expenditure was estimated adding parents’ reports of “household reported weekly expenditure on food items,” “monthly expenditure on services and short term goods,” and “other expenditures,” and was divided into a binary variable, based on a median split, to classify households into low and high consumption.

#### Adolescent psychosocial measures (adolescent self-report)

##### Perceived control

A modified version of the General Perceived Control (Mastery) scale (23), developed by Pearlin and Schooler (23) was used. It consists of seven items rated on a 4-point Likert scale from “strongly agree” to “strongly disagree.” Sample items include, “I have little control over the things that happen to me,” “There is little I can do to change many of the important things in my life,” “What happens to me in the future mostly depends on me,” and “I can do just about anything I really set my mind to do.” A summary score was used to create a dichotomous variable based on a median split: “low control” and “high control.”

##### Social support

Adolescents provided data on the total number of close friends they had and the number of close friends with whom they discussed personal problems. A summary score was used to create a binary variable based on a median split for “low” and “high” social support.

##### Team or group membership

Adolescents responded to the question, “do you belong to a sports or recreational team or group?” (yes/no).

#### Adolescent risk behaviors (adolescent self-report)

##### Substance use

Adolescents were asked if they currently smoke (yes/no), and the average number of beers and liquor they consume in a normal week. Drinking was defined by consuming >5 beers or shots of hard liquor in a normal week. Drinking with repercussions was defined as those who reported drinking alcohol, even if just occasionally, and who over the last 30 days had an occasion in which they failed to complete an activity, like going to school or work, as a result of their alcohol consumption (yes/no).

##### Sexual behavior

Adolescents reported if they had ever had sexual intercourse (yes/no). If yes, they were asked if they had used a condom during the last time they had sex (yes/no). To determine if they had ever participated in compensated sex, they were asked a series of questions regarding receipt of gifts from various sexual partners after they had had sex (coded yes for compensated sex if they had done so from any partner).

##### Deviant and aggressive behaviors

Adolescents were asked: (1) if they had ever been detained by the police (yes/no), and (2) how many physical altercations they had been in. The latter was dichotomized to distinguish adolescents who reported having ever been in a fight and those who had not.

##### Obesity-related behaviors

Adolescents were asked for the prior day how many bags of chips, packets of cakes or sweet breads and packets of sweets they consumed and how many sodas. Two binary variables were made: high/low junk food consumption and high/low soda consumption. Three or more pieces of junk food and three or more sodas were respectively used to distinguish high from low consumption. Regarding sedentary behavior, adolescents were asked the number of hours they watched television during their last viewing. Adolescents who reported viewing 4 h or more were classified as heavy television watchers, whereas those watching fewer than 4 h were reported as light television watchers. Adolescents were also asked the number of days they exercised during the previous week. A dichotomous variable was created, categorizing adolescents into those who reported ever having exercised during the previous week and those who did not.

### Statistical analysis

Statistical analyses were conducted using STATA 10.0 (STATA Corporation, College Station, TX). Descriptive statistics were generated; one-sample test of proportions was used to investigate sampling characteristics according to demographic, socioeconomic, and psychosocial factors and risk behaviors.

To examine the associations between adolescent *perceived social mobility* and risk behaviors, we conducted chi-square analyses. The difference in the proportion of adolescents who reported having adopted the behavior of interest was calculated according to whether they were classified as being “downwardly mobile” versus “stable” or “upwardly mobile.” Logistic regression analyses were conducted, examining the association between adolescent perceived social mobility and risk behaviors, controlling for objective SES. All analyses controlled for age, sex, dropout status, welfare status, fixed effect at the state level, and clustering at the neighborhood level.

The second aspect of the study, examining the association between *perceived social mobility* (dependent variable), and demographic, socioeconomic, and psychosocial characteristics (independent variables), used Pearson correlation analyses. Multiple logistic regression analysis was run, mutually controlling for adolescent demographic and socioeconomic characteristics (age, sex, paid work, school dropout), parental and household SES (e.g., maternal education, paternal education, total monthly household expenditures per person), psychosocial factors (e.g., perceived control, belonging to a group or team, social support), and other covariates, including welfare status (adolescents from recipient households of the Mexican government’s poverty alleviation program, “Oportunidades”), the fixed effect of state (adolescent residency in one of seven sample states in Mexico) and clustering at the neighborhood level.

## Results

### Sample characteristics

**Figure 2:**
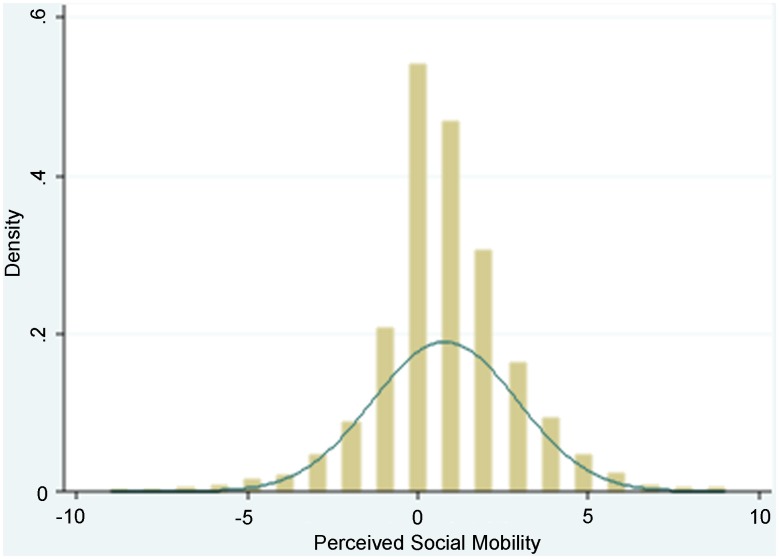
**Distribution of adolescent perceived social mobility scores (mean = 0.80, SD = 2.11) overlaid with a normal distribution**.

Adolescent *perceived social mobility* scores were approximately normally distributed (mean = 0.80, SD = 2.11) (Figure 2). The sample is described in Table 1. Just over half (54%) of the adolescents in the sample were classified as being upwardly mobile, defined as adolescents having a rank of one or more steps higher on the future ladder compared to the current ladder scale.

**Table 1:** **Study sample characteristics according to demographic, socioeconomic, psychosocial, and risk behavior factors^a^**.

Variable	# Of responses for given question (*n*)	Prevalence of given factor (%)
**Adolescent demographics and socioeconomic status**	5189	
Age (years)	16.85 (mean)	1.93 (SD^b^)
Sex (female = 1)		50.78
School dropout (yes = 1)		38.83
Paid job (yes = 1)		62.94
**Parental and household socioeconomic status indicators**	5189	
Maternal education (secondary and above = 1)		35.27
Paternal education (secondary and above = 1)		33.71
Monthly household expenditures (high consumption = 1)		50.38
**Psychosocial factors**	5189	
Perceived control (high = 1)		47.33
Team or group membership (yes = 1)		39.26
Social support (high = 1)		45.71
**Risk behaviors**		
Currently smoke (yes = 1)	5166	17.05
Excessive alcohol consumption (5 drinks or more = 1)	5189	7.15
Problem drinking (yes = 1)	1589	6.42
Sexually active (yes = 1)	5189	20.74
Condom use (yes = 1)	808	53.34
Compensated sex (yes = 1)	1068	9.46
Detained by police (yes = 1)	5167	7.93
Fight (2 or more = 1)	5167	35.16
Junk food consumption (3 or more = 1)	5189	22.86
Soda consumption (3 or more = 1)	5189	12.51
Television watching (4 h or more = 1)	5189	40.41
Exercise (yes = 1)	5189	49.68
**Adolescent perceived class identity**	5189	
Adolescent perceived social mobility (upward = 1)		54.33

*^a^One sample test of proportions were used to investigate the difference in percentage of the study sample with and without given descriptive characteristics*.

*^b^SDs*.

*Total monthly household expenditures per person, perceived control and social support are dichotomous variables created using a median split. Problem drinking, condom use, and compensated sex are all filter questions on the adolescent survey and therefore have a fewer number of responses*.

### Anticipated mobility and risk behaviors

Upward social mobility was significantly associated with a lower prevalence of drinking (*p* = 0.008), drinking with repercussions (*p* = 0.004), engaging in compensated sex (*p* = 0.002), being detained by the police (*p* = 0.002), getting into physical altercations (*p* = 0.007), being within the top twentieth percentile of junk food consumption (*p* = 0.002) and soda consumption (*p* = 0.001), and watching many hours of television (*p* < 0.0001). It was also significantly associated with a higher prevalence of condom use during last intercourse (*p* = 0.011) and doing more physical exercise (*p* < 0.0001) (Table 2). No significant associations were found between *perceived social mobility* and current tobacco smoking or with being sexually active.

**Table 2:** **Proportion (%) engaging in risk behavior according to perceived social mobility**.

Risk behavior variables	Sample size (*n*)	Society mobility		*p*-Value
	
		Downward/no change	Upward	
Currently smokes	5166	17.33	16.82	0.628
Excessive drinking (≥5 drinks)	5189	8.19	6.28	0.008
Problem drinks^a^	1589	8.38	4.81	0.004
Sexually active	5189	21.05	20.47	0.604
Used condom during last sexual intercourse	808	48.42	57.44	0.011
Had compensated sex	1068	12.40	6.94	0.002
Has been detained by police	5167	9.21	6.87	0.002
Fights (≥2)	5189	25.15	21.25	0.001
Junk food consumption (≥3)	5189	24.81	21.21	0.002
Soda consumption (≥3)	5189	14.22	11.07	0.001
Television watching (≥4 h)	5189	44.18	37.25	<0.0001
Exercises	5189	45.11	53.53	<0.0001

*Adolescents completed survey questions on risk behaviors and perceived social status (used to create the social mobility indicator) at the same time; all data is cross-sectional. Problem drinking, condom use, and compensated sex are filter questions on the adolescent survey and therefore have a fewer number of responses*.

*^a^Problem drinking refers to the adolescents who reported drinking alcohol, even if just occasionally, and who over the last 30 days had an occasion in which they failed to complete an activity, like going to school or work, as a result of their alcohol consumption (yes/no)*.

In adjusted logistic regression analyses (Tables 3 and 4), controlling for objective indicators of SES (parental education and household expenditures), and other covariates (age, sex, welfare status, the fixed effect of state and clustering at the community neighborhood level), adolescents who were classified as being upwardly mobile compared to those who were classified as being stable or being downwardly mobile are less likely to report drinking (OR = 0.81, 95% CI: 0.66–0.99), drinking with repercussions (OR = 0.63, 95% CI: 0.41–0.97), compensated sex (OR = 0.54, 95% CI: 0.34–0.86), police detainment (OR = 0.80, 95% CI: 0.67–0.96), getting into physical altercations (OR = 0.88, 95% CI: 0.77–0.99), eating ≥3 servings of junk food yesterday (OR = 0.83, 95% CI: 0.72 = 0.96), drinking ≥3 sodas yesterday (OR = 0.81, 95% CI: 0.68–0.96), and watching ≥4 h of television during their last viewing (OR = 0.79, 95% CI: 0.71–0.87). They were more likely to report exercising during the previous week (OR = 1.26, 95% CI: 1.14–1.40) and using a condom during last intercourse (OR = 1.45, 95% CI: 1.04–2.01).

**Table 3:** **Logistic regression analyses showing the cross-sectional associations between parental and household objective SES and adolescent perceived social mobility and risk behaviors associated with substance use, and sexual and delinquent behavior^a^ (Odds Ratios and Robust 95% Confidence Intervals presented)**.

Social status variables	Excessive drinking	Problem drinking	Condom use	Compensated sex	Police detainment	Physical fighting
Upward social mobility	0.81* (0.66–0.99)	0.63* (0.41–0.97)	1.45* (1.04–2.01)	0.54** (0.34–0.86)	0.80* (0.67–0.96)	0.88* (0.77–0.99)
High maternal education	1.09 (0.84–1.41)	0.88 (0.57–1.38)	1.45* (1.04–2.04)	0.96 (0.61–1.50)	1.1 (0.86–1.41)	1.08 (0.91–1.27)
High paternal education	1.12 (0.87–1.45)	0.74 (0.42–1.28)	0.85 (0.59–1.22)	1.26 (0.74–2.14)	0.82 (0.63–1.06)	0.83* (0.70–0.98)
High household expenditures	1.11 (0.89–1.38)	1.29 (0.76–2.19)	1.13 (0.85–1.50)	0.83 (0.53–1.31)	0.94 (0.76–1.15)	1.14 (0.99–1.32)
Observations	5189	1559	808	1048	5167	5189

**p < 0.01, *p < 0.05.

*^a^All models control for age, sex, dropout status, state, welfare status, and clustering at the community level*.

*No change in or downward social mobility = reference category for social mobility; no education through primary = reference category for maternal and paternal education; low = reference category for monthly household expenditures. Table does not include currently smokes and sexually active because there was no significant association between these risk behaviors and perceived social mobility*.

**Table 4:** **Logistic regression analyses showing the cross-sectional associations between parental and household objective SES and adolescent perceived social mobility and obesity-related risk behaviors (*n* = 5189)^a^ (Odds Ratios and Robust 95% Confidence Intervals presented)**.

Social status variables	Ate junk food	Drank soda	Watched TV	Exercised
Upward social mobility	0.83* (0.72–0.96)	0.81* (0.68–0.96)	0.79** (0.71–0.87)	1.26** (1.14–1.40)
High maternal education	0.85* (0.75–0.98)	0.98 (0.81–1.18)	0.9 (0.79–1.03)	1.01 (0.91–1.13)
High paternal education	0.95 (0.83–1.09)	0.82* (0.69–0.97)	0.93 (0.82–1.05)	1.17** (1.04–1.32)
High household expenditures	1.07 (0.94–1.23)	1.08 (0.92–1.26)	0.91 (0.81–1.02)	1.27** (1.09–1.49)

**p < 0.01, *p < 0.05.

*^a^All models control for age, sex, dropout status, state, welfare status, and clustering at the community level*.

*No change in or downward social mobility = reference category for social mobility; no education through primary = reference category for maternal and paternal education; low = reference category for monthly household expenditures*.

### Correlates of anticipated mobility

In Pearson correlation analyses (Table 5), *perceived social mobility* was significantly correlated with a greater likelihood of having parents with more than a primary education, greater monthly household expenditures per person, having greater perceived control, belonging to a team or being a member of a group, having more social support, and with a lower likelihood of having a paid job and dropping out of school. Age and sex were not significantly correlated with upward social mobility (data not shown). In the multiple logistic regression model (Table 2), controlling for covariates (age, sex, welfare status, the fixed effect of state and clustering at the community level), adolescents who were classified as being upwardly mobile, compared to those who were classified as being stable or downwardly mobile, were more likely to have stayed in school (OR = 0.61, 95% CI: 0.55–0.69) and to have greater perceived control (OR = 1.06, 95% CI: 1.69–2.12). No other characteristics remained significant in the adjusted analysis. There was no evidence of co-linearity among parental and household SES and adolescent SES variables.

**Table 5:** **Correlates of adolescent perceived upward social mobility (*n* = 5189)**.

Socioeconomic andpsychosocial variables	Pearson correlations (r)	Logistic regression (OR/95% CI)^a^
Maternal education (≥secondary = 1)	0.04**	0.98 (0.85–1.12)
Paternal education (≥secondary = 1)	0.04**	1.06 (0.94–1.20)
Monthly household expenditures (high = 1)	0.05**	1.09 (0.96–1.24)
Paid work (yes = 1)	−0.03*	1.02 (0.91–1.14)
School dropout (yes = 1)	−0.14**	0.61** (0.55–0.69)
Perceived control (high = 1)	0.18**	1.89** (1.69–2.12)
Team/group membership (yes = 1)	0.03*	1.06 (0.95–1.18)
Social support (high = 1)	0.04*	1.08 (0.96–1.21)

**p < 0.01, *p < 0.05.

*^a^The multiple logistic regression analysis reports odds ratios and robust 95% confidence intervals. The model controls for age, sex, welfare status, fixed effect of state, and clustering at the community level. No education through primary = reference category for maternal and paternal education; low = reference category for monthly household expenditures, perceived control and social support*.

## Discussion

This study set out to explore the usefulness of a new psychosocial indicator, *perceived social mobility*, for understanding risk behaviors during adolescence. This is the first study to introduce the concept of anticipated mobility, adolescents’ expected intergenerational transmission from disadvantage from their family of origin to their future nuclear family. We began by calculating the difference between each subject’s rankings of current and anticipated familial social status. This new and simple approach overcomes several methodological limitations that arise when measuring intergenerational social mobility with conventional objective indicators of social position. As adolescence is a period of social and economic transition, and social rank may therefore be a moving target, this new construct adds the important dimension of expectations. Our study demonstrates that *perceived social mobility* is significantly associated with multiple risk behaviors within the context of Mexican society, providing evidence for the usefulness of this new indicator in informing our understanding of the social gradient in health.

The data reveal that among adolescents in our sample who were classified as “upwardly mobile” on our scale of *perceived social mobility* (as compared to those classified as “stable” or “downwardly mobile,”) there is a significantly lower prevalence of excessive drinking, problem drinking, compensated sex, police detainment, physical altercations, consumption of large quantities of junk food and soda, and watching multiple hours of television during last viewing. Among this group, there is a significantly higher prevalence of exercising and condom use during last intercourse.

Our findings are consistent with other studies, which have found that adolescent expectations, including lack of perceived future opportunities, little perceived control and low levels of optimism, may put adolescents at-risk for adopting health-compromising behaviors (24–29). For example, lack of perceived future opportunities have been shown to be associated with teen pregnancy, substance use and juvenile crimes (25). Little sense of control over one’s life has been associated with non-use of contraception among females (27) and greater likelihood of initiating smoking (24). Low levels of optimism have been shown to be associated with an increased risk of overdosing (28), experiencing anger, a precursor of violent behavior (29), and being more stressed (26). There is also evidence that adolescents who are less hopeful about the future are more fatalistic (30).

Can previous research findings and theory help us to tease apart the causal directions for the significant cross-sectional associations that we found in this study? The research done by others suggests that sexual, obesity-related, deviant and substance use behaviors are more likely a consequence than a cause of anticipated social mobility. However, the social selection versus social causation debate around health has been a long and complicated one. According to social selection theory, individuals who are healthiest may be more likely to be upwardly mobile and more capable of moving up the social hierarchy and attaining high socioeconomic standing (31, 32). Alternatively, it has been argued that poor social and material circumstances increase disease risk, thereby producing a social gradient in health (2, 16). The results of our study may enhance the quality of this discourse. While some risk behaviors may indeed result from the anticipation of upward mobility, or lack thereof, the second part of our study suggests that it may be useful to consider what factors contribute to the optimism involved in believing in one’s ability to rise above current conditions.

We investigated the associations between adolescent *perceived social mobility* and multiple socioeconomic and psychosocial factors. Perceived control and school dropout status remained statistically significant in multiple logistic regression models. Both of these factors reflect current and future social and economic conditions, opportunities and life options of the young participants. Adolescents who drop out of school or do not believe they have much control over their destiny may accurately anticipate less upward mobility and thus be more likely to adopt risk behaviors. The importance of education for social mobility has been established in previous studies (33). That perceived control remained in the equation further points to the potentially important role of mastery and hope in the way adolescents determine their future social standing in relation to their current social standing.

### Limitations

There are some important limitations to note, each of which suggests directions for future research. The cross-sectional nature of our study precludes our ability to make causal inferences. While a growing body of research suggests the importance of subjective measures of adolescent social status in understanding the links between relative deprivation and health during adolescence, adolescent *perceived social mobility* is a new construct that has not yet been validated. Although audio computer-assisted self-interviews were used to solicit honest responses, adolescent self-report of risk behaviors could still be biased, particularly for the sensitive questions involving substance use and sexual, deviant and aggressive behaviors. Our study findings cannot be generalized to adolescents outside of Mexico or not living in extreme poverty.

## Conclusion

Our findings suggest the importance of adolescent *perceived social mobility*, a new psychosocial indicator that measures adolescent predictions of their socioeconomic trajectory. This easily assessed component of adolescent relative deprivation suggests the importance of future expectations, a dimension of hope, in understanding the social gradient in adolescent health. Future research into the associations between *perceived social mobility*, hope, optimism, and the inequality of opportunity in general, will help determine the role of psychosocial and structural factors in adolescent risk behaviors. Future research should examine these associations among diverse national and socioeconomic adolescent and young adult cohorts, including disadvantaged youth. Whether adolescent *perceived social mobility* explains differentials in adult health remains to be determined. This research suggests the possibility of implementing policies and interventions that provide adolescents with real reasons to be hopeful about their trajectories.

## Conflict of Interest Statement

The authors declare that the research was conducted in the absence of any commercial or financial relationships that could be construed as a potential conflict of interest.

## References

[1] Link BG Phelan J. Social conditions as fundamental causes of disease. J Health Soc Behav (1995) Spec No:80–94. 10.2307/2626958 7560851

[2] Marmot MG Wilkinson RG Social Determinants of Health. Oxford, NY: Oxford University Press (2006).

[3] Brooks-Gunn J Duncan GJ The effects of poverty on children. Future Child (1997) 7(2):55–71 10.2307/1602387 9299837

[4] Evans GW Kim P. Childhood poverty and health: cumulative risk exposure and stress dysregulation. Psychol Sci (2007) 18(11):953–7. 10.1111/j.1467-9280.2007.02008.x 17958708

[5] Starfield B Riley AW Witt WP Robertson J Social class gradients in health during adolescence. J Epidemiol Community Health (2002) 56(5):354–61 10.1136/jech.56.5.354 11964432 PMC1732142

[6] West P. Health inequalities in the early years: is there equalisation in youth? Soc Sci Med (1997) 44(6):833–58. 10.1016/S0277-9536(96)00188-8 9080566

[7] West P Sweeting H. Evidence on equalisation in health in youth from the West of Scotland. Soc Sci Med (2004) 59(1):13–27. 10.1016/j.socscimed.2003.12.004 15087139

[8] Goodman E Adler NE Daniels SR Morrison JA Slap GB Dolan LM. Impact of objective and subjective social status on obesity in a biracial cohort of adolescents. Obes Res (2003) 11(8):1018–26. 10.1038/oby.2003.140 12917508

[9] Goodman E Adler NE Kawachi I Frazier AL Huang B Colditz GA. Adolescents’ perceptions of social status: development and evaluation of a new indicator. Pediatrics (2001) 108(2):E31. 10.1542/peds.108.2.e31 11483841

[10] Goodman E Huang B Schafer-Kalkhoff T Adler NE. Perceived socioeconomic status: a new type of identity that influences adolescents’ self-rated health. J Adolesc Health (2007) 41(5):479–87. 10.1016/j.jadohealth.2007.05.020 17950168 PMC2204090

[11] Gruenewald TL Kemeny ME Aziz N. Subjective social status moderates cortisol responses to social threat. Brain Behav Immun (2006) 20(4):410–9. 10.1016/j.bbi.2005.11.005 16412608

[12] Finkelstein DM Kubzansky LD Goodman E. Social status, stress, and adolescent smoking. J Adolesc Health (2006) 39(5):678–85. 10.1016/j.jadohealth.2006.04.011 17046504

[13] Ritterman ML Fernald LC Ozer EJ Adler NE Gutierrez JP Syme SL. Objective and subjective social class gradients for substance use among Mexican adolescents. Soc Sci Med (2009) 68(10):1843–51. 10.1016/j.socscimed.2009.02.048 19342140

[14] Adler NE Newman K. Socioeconomic disparities in health: pathways and policies. Health Aff (Millwood) (2002) 21(2):60–76. 10.1377/hlthaff.21.2.60 11900187

[15] Goodman E Amick BC Rezendes MO Levine S Kagan J Rogers WH Adolescents’ understanding of social class: a comparison of white upper middle class and working class youth. J Adolesc Health (2000) 27(2):80–3. 10.1016/S1054-139X(99)00116-0 10899467

[16] Dahl E Social mobility and health: cause or effect? BMJ (1996) 313(7055):435–6 10.1136/bmj.313.7055.435 8776298 PMC2351864

[17] Manor O Matthews S Power C. Health selection: the role of inter- and intra-generational mobility on social inequalities in health. Soc Sci Med (2003) 57(11):2217–27. 10.1016/S0277-9536(03)00097-2 14512251

[18] Glendinning A Shucksmith J Hendry L. Social class and adolescent smoking behaviour. Soc Sci Med (1994) 38(10):1449–60. 10.1016/0277-9536(94)90283-6 8023194

[19] Karvonen S Rimpela AH Rimpela MK. Social mobility and health related behaviours in young people. J Epidemiol Community Health (1999) 53(4):211–7. 10.1136/jech.53.4.211 10396546 PMC1756866

[20] Pulkki L Kivimaki M Elovainio M Viikari J Keltikangas-Jarvinen L. Contribution of socioeconomic status to the association between hostility and cardiovascular risk behaviors: a prospective cohort study. Am J Epidemiol (2003) 158(8):736–42. 10.1093/aje/kwg204 14561662

[21] Ball K Mishra GD. Whose socioeconomic status influences a woman’s obesity risk: her mother’s, her father’s, or her own? Int J Epidemiol (2006) 35(1):131–8. 10.1093/ije/dyi216 16284404

[22] Brunner E Shipley MJ Blane D Smith GD Marmot MG. When does cardiovascular risk start? Past and present socioeconomic circumstances and risk factors in adulthood. J Epidemiol Community Health (1999) 53(12):757–64. 10.1136/jech.53.12.757 10656084 PMC1756821

[23] Pearlin LI Schooler C The structure of coping. J Health Soc Behav (1978) 19(1):2–21 10.2307/2136539 649936

[24] Clarke JH MacPherson BV Holmes DR Cigarette smoking and external locus of control among young adolescents. J Health Soc Behav (1982) 23(3):253–9 10.2307/2136633 7175161

[25] Dempsey PL Dealing with teen-age pregnancy. Bull N Y Acad Med (1991) 67(1):26–9. 2009415 PMC1809800

[26] Finkelstein DM Kubzansky LD Capitman J Goodman E. Socioeconomic differences in adolescent stress: the role of psychological resources. J Adolesc Health (2007) 40(2):127–34. 10.1016/j.jadohealth.2006.10.006 17259052 PMC1847603

[27] Kowaleski-Jones L Mott FL. Sex, contraception and childbearing among high-risk youth: do different factors influence males and females? Fam Plann Perspect (1998) 30(4):163–9. 10.2307/2991677 9711453

[28] O’Connor RC Rasmussen S Miles J Hawton K. Self-harm in adolescents: self-report survey in schools in Scotland. Br J Psychiatry (2009) 194(1):68–72. 10.1192/bjp.bp.107.047704 19118330

[29] Puskar K Ren D Bernardo LM Haley T Stark KH. Anger correlated with psychosocial variables in rural youth. Issues Compr Pediatr Nurs (2008) 31(2):71–87. 10.1080/01460860802023513 18569198 PMC2771611

[30] Jacobson LD Wilkinson CE. Review of teenage health: time for a new direction. Br J Gen Pract (1994) 44(386):420–4. 8790657 PMC1238994

[31] Blane D Smith GD Bartley M Social selection: what does it contribute to social class differences in health? Soc Health and Illn (1993) 15(1):1–15 10.1111/j.1467-9566.1993.tb00328.x

[32] West P. Rethinking the health selection explanation for health inequalities. Soc Sci Med (1991) 32(4):373–84. 10.1016/0277-9536(91)90338-D 2024152

[33] Hagquist CE. Health inequalities among adolescents: the impact of academic orientation and parents’ education. Eur J Public Health (2007) 17(1):21–6. 10.1093/eurpub/ckl087 16777839

